# Decision‐making and risk‐taking as predictors of health risk behaviors in the Millennium Cohort Study

**DOI:** 10.1002/jcv2.70071

**Published:** 2025-12-09

**Authors:** Nicole G. Hammond, Georgia Condran, Elise E. DeVito, Marie‐Claude Geoffroy, Ian Colman

**Affiliations:** ^1^ School of Epidemiology and Public Health University of Ottawa Ottawa Ontario Canada; ^2^ Temerty Faculty of Medicine University of Toronto Toronto Ontario Canada; ^3^ Department of Psychiatry Yale School of Medicine New Haven Connecticut USA; ^4^ Department of Educational and Counselling Psychology McGill University Montreal Québec Canada; ^5^ Department of Psychiatry McGill University & Douglas Mental Health University Institute Montreal Québec Canada; ^6^ Centre for Fertility and Health Norwegian Institute of Public Health Oslo Norway

**Keywords:** adolescence, cohort study, gambling task, health behaviors, risk‐taking

## Abstract

**Background:**

Facets of decision‐making and risk‐taking are implicated in adolescent health risk behaviors; however, whether they may lead to adolescent engagement in substance use, gambling, and self‐harm is unknown.

**Methods:**

We used the Millennium Cohort Study to test whether a task‐based measure of decision‐making and risk‐taking predicts engagement in adolescent health risk behaviors. Participants were born in the United Kingdom (2000–2002) and surveyed in‐home at ages 14 (*n* = 10,531) and 17 (*n* = 8417). A computerized task‐based measure of decision‐making and risk‐taking for reward (Cambridge Gambling Task) measured impulsivity, quality of decision‐making, risk adjustment, and risk‐taking (exposures) at age 14. Several health risk behaviors (outcomes) were self‐reported at 14/17 years: cigarette use, electronic cigarette/vaping use, drinking, cannabis use, other illegal drug use (e.g., ecstasy), gambling, and self‐harm. We conducted adjusted multinomial and logistic regression models.

**Results:**

Computerized task‐based measures of greater impulsivity and risk‐taking were most consistently associated with self‐reported health risk behaviors at 14 and 17 years. Better quality of decision‐making and risk adjustment were inconsistently associated with health outcomes at age 14; however, better risk adjustment was related to a reduced likelihood of all levels of cigarette and e‐cigarette/vaping use (e.g., occasionally/regularly) when compared to nonusers. At age 14, risk‐taking was associated with every self‐reported health risk behavior (e.g., substance use, gambling) except for self‐harm. In prospective models, relationships were attenuated, but risk‐taking predicted new onset engagement in all forms of substance use except alcohol consumption and self‐harm. Risk‐taking was most strongly associated with other drug use (age 14: odds ratio (OR) = 11.26, 95% CI: 1.48, 86.01) and predictive of former vaping use (age 17: OR = 4.10, 95% CI: 1.43, 11.76).

**Conclusion:**

Risky betting on a computerized risk‐taking task appears highly indicative of substance use and recent gambling at age 14 and predictive of new onset substance use and gambling 3 years later (age 17) for both sexes, but not self‐harm.

## Key Points

**What's known?**

- Differences in decision‐making and risk‐taking may precede engagement in adolescent health risk behaviors. Longitudinal, population‐based studies of task‐based measures of decision‐making and risk‐taking are needed.

**What's new?**

- Using a computerized task‐based measure of decision‐making and risk‐taking for reward (Cambridge Gambling Task [CGT]), we examined relationships with health risk behaviors cross‐sectionally (age 14) and longitudinally (age 17) in a birth cohort.
- Risky betting was associated with substance use and gambling at age 14 and strongly predicted new‐onset substance use and gambling behavior at age 17 for both sexes.
- Self‐harm was not statistically related to any of the subtasks measured by the CGT: impulsivity, risk adjustment, quality of decision‐making, or risk‐taking.

**What's relevant?**

- Behavioral risk‐taking, as captured by the CGT, may hold clinical utility in predicting increased risk of adolescent substance use and gambling behavior.

## INTRODUCTION

Substance use, self‐harm, and gambling are health risk behaviors and major public health issues in adolescence. Substance use and self‐harm in adolescence and emerging adulthood are independently tied to premature mortality (Clark et al., 2008), while gambling, an increasingly recognized adolescent‐related public health issue (Armitage, 2021), is as well (Karlsson & Håkansson, 2018). Evidence of the consequences of problematic gambling behavior in adolescence is mounting, including recent work demonstrating that worsening severity is linked to future risk of suicide attempt among those aged 16–24 (Wardle et al., 2023). Vulnerabilities in decision‐making and risk‐taking may affect adolescent engagement in behaviors that may pose health risks (Balogh et al., 2013; Casey et al., 2008). Neurobiological delays in the maturation of prefrontal neural systems may partly explain adolescent engagement in risky or adverse health behaviors (Balogh et al., 2013). An increased sensitivity to reward at a time when impulse control is still developing may also play a role (Casey et al., 2008). Regardless, adolescence is a developmental period characterized by complexity, vulnerability, and potential risk of harm or death due to engagement in health risk behaviors (Dahl et al., 2018). What is not clearly understood is how task‐based measures of decision‐making and risk‐taking in adolescence may predict real‐world engagement in health risk behaviors which may pose a threat to healthy development.

More specifically, longitudinal, population‐based studies of how validated task‐based measures may prospectively predict and map onto actual engagement in adolescent risky health behaviors are lacking. In other words, do task‐based measures have real‐world applications for adolescents (Balogh et al., 2013)? To date, one relevant study examined an objective, standardized measure of decision‐making in relation to substance use and self‐harm in adolescence (Hosozawa et al., 2021). While the authors examined differences between adolescents diagnosed with autism spectrum disorder and typically developing youth, they observed associations between age 14 decision‐making, illegal drug use, and self‐harm, independent of neurodevelopmental diagnosis and earlier (age 11) decision‐making performance (Hosozawa et al., 2021). However, impulsivity and risk‐taking, psychological constructs heavily implicated in addictions and mental health, were not of focus.

Other studies relating behavioral measurement of risk‐taking and decision‐making with adverse health behaviors among young people focus on emerging adulthood (18–29 years; Chamberlain et al., 2013; Goudriaan et al., 2011; Grant et al., 2011; Harvanko et al., 2013), neglecting adolescence (10–19 years), when experimentation and risky health behavior engagement usually commences and becomes prevalent. Longitudinally, poorer (Goudriaan et al., 2011) and riskier (Harvanko et al., 2013) decision‐making predict short‐term (1–2 years) problematic alcohol use. Cross‐sectionally, differences in decision‐making and risk‐taking are documented among emerging adults who experience suicidality (Chamberlain et al., 2013), are at risk for problematic gambling (Grant et al., 2011), and among adolescents with potentially problematic substance use (Schneider et al., 2012). Population‐representative investigation during adolescence is needed to confirm prospective relationships between cognitive markers of decision‐making and negative health behaviors.

As such, study objectives were to determine whether scores on a task‐based gambling test at age 14 can predict substance use, gambling, and self‐harm 3 years later, addressing a critical gap in the literature. We also sought to explore the potential for sex differences. Males and females appear to have functional differences in the neural networks tested by behavioral measures (Gaillard et al., 2021b), and there may be differential task‐based performance between sexes (Gaillard et al., 2021a). Potential sex‐related differences require attention (Gaillard et al., 2021b) and have been poorly documented to date. We hypothesized that scores on a task‐based measure of decision‐making and risk‐taking, the Cambridge Gambling Task (CGT; Rogers et al., 1999), would predict cross‐sectional and longitudinal engagement in health risk behaviors in adolescence.

Precisely, it was hypothesized that adverse behavioral scores (i.e., greater impulsivity and risk‐taking) would predict greater engagement in health risk behaviors. Meanwhile, positive behavioral scores (i.e., better quality of decision‐making and risk adjustment) would protect against engagement in negative health behaviors. Finally, not only has heterogeneity affected the ability of meta‐analyses to establish whether there exist overall sex differences across behavioral measures (Gaillard et al., 2021a, 2021b), but adolescent sex‐specific differences in the CGT have yet to be studied beyond an initial investigation into their relationship with emotional and depressive symptoms (Lewis et al., 2021). While this early work suggests that adolescent males may exhibit greater differences on some subtasks of the CGT (Lewis et al., 2021), this area of scientific inquiry is in its infancy, and caution should be used in drawing parallels from adult work given developmental and maturational differences between adult and youth populations. Thus, the secondary aim to examine potential sex‐related differences was exploratory.

## MATERIALS AND METHODS

### Data source

We used data from the Millennium Cohort Study (MCS), a large epidemiological survey of children born in the United Kingdom (UK) between 2000 and 2002 (Connelly & Platt, 2014). Families were enrolled when the eligible child was 9 months (Sweep 1; 2001) and followed prospectively. The nearly universal Child Benefit was used to identify eligible children, and the sampling method used a stratified, clustered design with economically disadvantaged and ethnic minority concentrated geographic areas oversampled (Plewis, 2007). Excluded from the Child Benefit were families with temporary or uncertain residency status (e.g., “members of foreign armed forces” or “asylum seekers”; Plewis, 2007). We used 2015 (age 14, Sweep 6) and 2018 (age 17, Sweep 7) data, permitting cross‐sectional (14 years) and longitudinal investigations (14–17 years). The survey response rates were 76.3% (2015; Centre for Longitudinal Studies, UCL Institute of Education, 2017) and 73.4% (2018; Centre for Longitudinal Studies, UCL Institute of Education, 2019), respectively. Before granting data access, the UK Data Service (www.ukdataservice.ac.uk/) approved this project, including the analytic plan.

### Decision‐making and risk‐taking (exposure)

The CGT (Rogers et al., 1999) is a measure of “decision‐making and risk‐taking under explicit risk conditions” (Wilson & Vassileva, 2018). The CGT is one of two tests from the Cambridge neuropsychological test automated battery administered to the cohort during an in‐home assessment at 14 years (Atkinson, 2015). The CGT was administered in a standardized format by trained staff using computer‐assisted personal interviews on a tablet. The presentation of the CGT is extensively described in detail elsewhere (DeVito et al., 2008; Wilson & Vassileva, 2018), including for the participants studied here (Atkinson, 2015). In short, participants were presented with a row of 10 boxes across the top of the tablet screen, with varying ratios of red to blue boxes (e.g., 6:4, 8:2). Each trial began with one box containing a yellow token hidden behind it. Participants pressed the “red” or “blue” button at the bottom of the screen to indicate which color box(es) they thought held the yellow token (the decision stage). The changing ratios of colored boxes (e.g., 6:4, 8:2) explicitly indicate the odds of the yellow token being hidden behind a red or blue box (i.e., whichever color is in the majority) (Wilson & Vassileva, 2018).

In the subsequent gambling stage(s), participants started with 100 points of no monetary value and were instructed to bet a proportion of their total available points. The amount of points wagered was determined based on their perceived confidence in their selected location (behind a red or blue box) of the yellow token. They were also informed that the goal was to obtain as many points as possible and that points would be added to or subtracted from their points total based on whether they won or lost. The percentage of points that a participant could bet (5%, 25%, 50%, 75%, or 95% of their current points total) was first presented in ascending then descending order, each time displayed for 5 s. The participant could adjust their bet by following the on‐screen prompts to select their chosen “stake box” (e.g., 25%) (Atkinson, 2015). Participants were notified whether they won or lost after each trial. The CGT takes ≤18 min to administer and produces several measures.

We studied the four subtask measures we considered most likely to be pertinent to our health outcomes: delay aversion or *impulsivity* (higher scores represent a tendency to bet more quickly or impulsively, with the amount of the bet influenced by its order of presentation [risk‐taking score in descending trials—risk‐taking score in ascending trials]), *quality of decision‐making* or rational decision‐making (average proportion of trials where the favored outcome was selected), *risk adjustment* (tendency to bet a higher proportion of points, on average, when the odds are better [i.e., more boxes of the favored color are presented] and to bet less when the odds are worse, which is an adjustment of betting behavior based on risk), and *risk‐taking* (average amount of points gambled regardless of the odds of winning in trials where the participant selected the favored outcome, with larger bets suggestive of greater risk preference [i.e., risk taking for reward]) (Atkinson, 2015; DeVito et al., 2008; Wilson & Vassileva, 2018). Higher scores indicate more of the measured behavior. For the interested reader, Atkinson (2015) presents in detail the calculation of outcome measures, and a visual representation of how a participant progresses through the CGT is available in Romeu et al. (2020).

### Health risk behaviors (outcomes)

Study outcomes (substance use, gambling, and self‐harm) were collected as part of adolescent‐reported risky behavior modules at ages 14/17. Outcome categories were the same at both ages (14/17) unless otherwise noted. We studied six forms of *substance use*: cigarette smoking, e‐cigarette use/vaping, ever‐drinking alcohol, binge drinking, cannabis use, and other illegal drug use. For *cigarette smoking*, we categorized participants as never smokers (tried smoking ≤ 1 time [referent]), former smokers (used to smoke but not now), and any cigarette use (i.e., occasional/regular smokers). *E‐cigarette use/vaping* was operationalized similarly: nonvapers (age 14: never used and age 17: ≤1 time [referent]), former vapers (used to but don't now), and any vaping use (i.e., occasional/regular vapers). Participants were asked whether they had ever consumed more than a few sips of an alcoholic drink (i.e., *ever drank alcohol* [yes/no (referent)]). If yes, they were subsequently asked to report on *binge drinking*. More specifically, whether they “ever had five or more alcoholic drinks at a time? A drink is half a pint of lager, beer or cider, one alcopop, a small glass of wine, or a measure of spirits” (yes/no [referent]). Finally, participants reported whether they had ever tried *cannabis* or *other illegal drugs* (e.g., ecstasy, cocaine, speed). Respondents who positively endorsed cannabis or other illegal drug use were modeled as having used the respective drug (yes) versus not (referent).

Besides measures of substance use, we also included *gambling* and *self‐harm*. Gambling was assessed using one question: “Have you spent any of your *own money* on any of the following in the past 4 weeks? We want to know about games you played yourself.” Participants were prompted to report whether they used “fruit machines,” placed “a private bet for money (e.g., with friends),” placed “a bet at a betting shop (e.g., on football or horseracing),” or engaged in “any other gambling.” Respondents were considered to have gambled if they positively endorsed one or more of the four forms of gambling versus not (referent). While self‐harm was captured at 14/17 years, it was measured differently. At age 14, respondents were asked about nonspecific (with or without suicidal intent) self‐harm: “In the past year have you hurt yourself on purpose in any way?” (yes/no [referent]). In contrast, at age 17, respondents were asked “Have you ever hurt yourself on purpose in an attempt to end your life?” (yes/no), and “During the last year, have you hurt yourself on purpose in any of the following ways?” (yes/no for each): “cut or stabbed yourself,” “burned yourself,” “bruised or pinched yourself,” “taken an overdose of tablets,” “pulled out your hair”,” and “hurt yourself some other way.” Using both items at age 17, we differentiated between respondents with no self‐harm (referent), non‐suicidal self‐harm (engaged in past‐year self‐harm but never made a suicide attempt), and (ever) suicide attempt. Elaborations on the conceptualization of the outcomes are presented in Supporting Information S1: Appendix S1, and question wording in Supporting Information S1: Appendix S2 (see Table S1).

### Confounders

We adjusted for child‐level and family‐level confounders measured at age 14. For the child, we included sex (female/male [referent]), age (continuous), self‐reported race/ethnicity (White [referent], Mixed, Pakistani and Bangladeshi, and Other [e.g., Indian, Black or Black British]), pubertal status (continuous), internalizing symptoms (continuous), and crystallized intelligence (continuous) (Supporting Information S1: Appendix S1). At the family level, we adjusted for household poverty (<60% of the median household income for the United Kingdom vs. not [referent]), and the educational and vocational attainment of the person most knowledgeable about the child (General Certificate of Secondary Education levels [referent], BA degree+, and other qualifications [overseas only]; Supporting Information S1: Appendix S1).

### Sample derivation

In cross‐sectional analyses, we included participants who completed the CGT (*n* = 10,710) and the victimization and risky behaviors module (self‐administered during in‐home assessment: *n* = 10,531) at age 14. For longitudinal models, we also required participation (*n* = 8674; retention: 82.4%) and completion of the risky behaviors module (self‐administered during in‐home assessment: *n* = 8417) at age 17 for all outcomes except gambling, which was captured in the self‐complete online questionnaire post‐in‐home assessment (*n* = 5848).

### Statistical analyses

We employed multivariable multinomial and logistic regression models to estimate odds ratios (OR) and corresponding 95% confidence intervals (95% CI) for our categorical and dichotomous health risk behavior outcomes, respectively, in which decision‐making and risk‐taking were the independent variables and health risk behaviors were the dependent variables. To explore whether sex was an effect modifier of the associations of interest, we introduced an interaction term (sex by exposure [e.g., impulsivity]). When there was a statistically significant interaction term (*p* < .05), we assessed improvement in model fit using the (−2) log‐likelihood test to compare fit indices between nested models with and without the inclusion of the interaction term (Supporting Information S1: Appendix S2). In the presence of effect measure modification, models were stratified by sex; otherwise, we adjusted for sex by including it as a confounder. We applied population‐level survey weights to account for the complex survey design and attrition. Cross‐sectional models were specific to age 14. In prospective models with participants followed longitudinally from 14 to 17 years, we modeled new onset health risk behaviors. To do so, we removed participants who positively endorsed the respective health risk behavior at age 14 to model new onset behavior (outcome) at age 17. We conducted analysis‐wise deletion to preserve the maximum sample size for each analysis. Thus, the sample sizes vary according to the analytic model and are noted in the respective table(s). We used SAS version 9.4 (SAS Institute Inc.).

### Ethics approval and data sharing

The University of Ottawa Research Ethics Board (File #H‐11‐21‐7644) approved this study. Approved project users can access study data through the UK Data Service. Adolescent and parent/caregiver participants of the MCS provided informed consent.

## RESULTS

### Descriptive

The cross‐sectional and longitudinal samples had nearly an equal split of males and females (Table 1). Most participants were White (≥80%), did not live in poverty (≥66.7%), and had a parent/caregiver (person most knowledgeable about the child) hold academic and vocational qualifications consistent with National Vocational Qualification standards (≥84.5%). As expected, the longitudinal sample had a smaller proportion of families living in poverty (21.1% longitudinal vs. 33.2% cross‐sectional), with more highly educated persons most knowledgeable about the child (National Vocational Qualifications levels 4–5: 48.1% longitudinal and 37.6% cross‐sectional).

**TABLE 1:** Characteristics of respondents and families in the Millennium Cohort Study.

	Cross‐sectional sample (*n* = 10,531)		Prospective longitudinal sample (*n* = 8417)	
	Unweighted *n*	Weighted %^a^	Unweighted *n*	Weighted %^a^
Child characteristics				
Sex				
Male	5190	51.3	4064	49.6
Female	5341	48.7	4353	50.4
Missing	0	–	0	–
Race/ethnicity				
White	8315	80.2	6618	84.4
Mixed	491	5.3	392	5.0
Pakistani/Bangladeshi	778	4.9	650	4.0
Other	857	8.6	697	5.8
Missing	90	1.0	60	0.8
Age				
*M* (SD)	10,527	14.26 (0.34)	8415	14.25 (0.31)
Missing	4	–	2	–
Pubertal status				
*M* (SD)	10,480	2.82 (0.56)	8387	2.83 (0.50)
Missing	51	–	30	–
Internalizing symptoms				
*M* (SD)	10,243	3.91 (3.48)	8202	3.63 (3.04)
Missing	288	–	215	–
Crystallized intelligence				
*M* (SD)	10,332	6.93 (2.55)	8260	7.29 (2.37)
Missing	199	–	157	–
Impulsivity				
*M* (SD)	10,527	0.27 (0.21)	8414	0.26 (0.19)
Missing	4	–	3	–
Quality of decision‐making				
*M* (SD)	10,531	0.88 (0.13)	8417	0.89 (0.11)
Missing	0	–	0	–
Risk adjustment				
*M* (SD)	10,530	0.98 (0.96)	8417	1.06 (0.88)
Missing	1	–	0	–
Risk‐taking				
*M* (SD)	10,530	0.52 (0.15)	8417	0.52 (0.13)
Missing	1	–	0	–
Family characteristics				
Poverty				
No	7510	66.7	6208	78.9
Yes	3011	33.2	2200	21.1
Missing	10	0.1	9	0.1
Parental education				
NVQ level 1–3	4490	46.9	3441	42.9
NVQ level 4–5	4763	37.6	4029	48.1
Other	1235	14.8	915	8.5
Missing	43	0.6	32	0.5

*Note*: Percentages may not total 100% due to rounding errors.

Abbreviation: NVQ, National Vocational Qualifications.

^a^ Weighted percentages are reported unless otherwise noted for continuous variables (mean [*M*] and standard deviation [SD]).

At age 14, drinking alcohol (48.1%), self‐harm (15.5%), and gambling (12.2%) were the most prevalent health risk behaviors (Table 2). E‐cigarette use/vaping was also common when including former e‐cigarette users/vapers, hereafter referred to as vapers (former vapers: 14.0%, occasional/regular vapers: 3.6%). The most common new‐onset health risk behaviors (i.e., behaviors not engaged in at age 14 but positively self‐reported at 17 years) were drinking alcohol (84.3%), binge drinking (56.5%), cannabis use (30.9%), and combined self‐harm (non‐suicidal self‐harm: 18.2%, suicide attempt: 7.5%; Table 2). Over time, engagement in health risk behaviors became more common, as evidenced by the increasing proportions of youth self‐reporting every health risk behavior at age 17. The age 17 proportion of cigarette smokers and e‐cigarette/vaping users was also high when including former smokers/vapers (28.4% [cigarette smoking] and 23.1% [e‐cigarette/vaping use]). Here, in the longitudinal models, former cigarette smokers and e‐cigarette/vaping users captured those who initiated use after age 14 and achieved cessation by 17 years.

**TABLE 2:** Prevalence of health risk behaviors among respondents in the Millennium Cohort Study.

	Cross‐sectional sample (14 years) *n* = 10,531		Prospective longitudinal sample (14–17 years) *n* = 8417	
	Unweighted *n*	Weighted %^a^	Unweighted *n*	Weighted %^a^
Cigarette smoking				
Nonsmokers	9822	91.5	6168	71.1
Former smokers	264	2.9	564	6.9
Occasional/regular smokers	378	4.7	1643	21.5
Missing	67	0.8	42	0.6
E‐cigarettes/vaping				
Nonvapers	8876	81.7	6474	76.3
Former vapers	1279	14.0	944	11.6
Occasional/regular vapers	316	3.6	955	11.5
Missing	61	0.8	44	0.6
Drank alcohol				
No	5889	51.6	1780	15.6
Yes	4617	48.1	6626	84.3
Missing	25	0.3	11	0.1
Binge drink				
No	3618	37.3	2235	27.8
Yes	985	10.7	4382	56.5
Not applicable	5914	51.9	1791	15.7
Missing	14	0.1	9	0.1
Cannabis use				
No	10,035	94.1	5998	68.8
Yes	464	5.5	2390	30.9
Missing	32	0.4	29	0.4
Other drug use				
No	10,433	98.8	7611	89.4
Yes	66	0.8	749	9.9
Missing	32	0.4	57	0.7
Any gambling^b^				
No	9249	87.1	5008	86.0
Yes	1226	12.2	816	13.7
Missing	56	0.7	24	0.3
Any self‐harm (14 years)			–	–
No	8929	83.9		
Yes	1537	15.5		
Missing	65	0.6		
Self‐harm (17 years)	–	–		
No			6257	73.8
Non‐suicidal self‐harm			1488	18.2
Suicide attempt			611	7.5
Missing			61	0.6

*Note*: Percentages may not total 100% due to rounding errors.

Abbreviation: NVQ, National Vocational Qualifications.

^a^ Weighted percentages are reported unless otherwise noted for continuous variables (mean [*M*] and standard deviation [SD]).

^b^ The longitudinal sample for gambling is smaller (*n* = 5848) because the measure was captured via a self‐complete online (web‐based) questionnaire after the in‐home assessment.

### Missing data and losses to follow‐up

We compared respondents with and without missing data on sex and quality of decision‐making; the measures without missing data at age 14. There were no statistical differences between participants with missing data on the exposures and confounders for sex (age 14, *p* = .267; age 14–17, *p* = .488) or quality of decision‐making (age 14, *p* = .291; age 14–17, *p* = .756). A greater proportion of those lost to follow‐up were male (11.6%) than female (9.0%, *p* < .001), and those lost to follow‐up had lower quality of decision‐making (*M* = 0.86) than those retained (*M* = 0.89, *p* < .001).

### Age 14 (cross‐sectional) models

At age 14, increasing risk‐taking was the CGT subtask most consistently associated with concurrent engagement in health risk behaviors (Table 3). More specifically, increasing risk‐taking as captured by a task‐based measure of risk‐taking for reward was associated with engagement in all adverse health outcomes at age 14, except for self‐harm (OR = 1.39, 95% CI: 0.79, 2.44, *p* = .260). The strongest relationships were for occasional/regular cigarette smokers (OR = 9.35, 95% CI: 3.11, 28.08, *p* < .001) and other drug use (OR = 11.26, 95% CI: 1.48, 86.01, *p* = .020). While there was evidence of effect modification by sex for risk‐taking (Supporting Information S1: Table S2), the only subtask at age 14 for which there was statistical support for effect measure modification, stratified results were unstable (Greenland et al., 2016). A sensitivity analysis applying Firth's bias correction (penalized regression; Firth, 1993) for rare events did not sufficiently address our concerns; as such, we report non‐sex‐stratified findings for risk‐taking (above).

**TABLE 3:** Cross‐sectional associations between task measures (impulsivity, quality of decision‐making, risk adjustment, and risk‐taking) of the Cambridge Gambling Task and health risk behaviors at age 14.

	Impulsivity		Quality of decision‐making		Risk adjustment		Risk‐taking	
	OR (95% CI)	*p*	OR (95% CI)	*p*	OR (95% CI)	*p*	OR (95% CI)	*p*
Cigarette smoking								
Nonsmokers	Referent		Referent		Referent		Referent	
Former smokers	1.67 (0.84, 3.32)	.142	0.33 (0.11, 1.00)	.051	**0.78 (0.66, 0.91)**	**.002**	**4.39 (1.34, 14.36)**	**.015**
Occasional/regular smokers	**3.15 (1.62, 6.13)**	**.001**	**0.27 (0.10. 0.75)**	**.012**	**0.85 (0.74, 0.98)**	**.020**	**9.35 (3.11, 28.08)**	**<.001**
E‐cigarettes/vaping								
Nonvapers	Referent		Referent		Referent		Referent	
Former vapers	1.25 (0.82, 1.89)	.305	**0.41 (0.24, 0.70)**	**.001**	**0.89 (0.83, 0.97)**	**.005**	**2.70 (1.49, 4.88)**	**.001**
Occasional/regular vapers	**2.74 (1.30, 5.75)**	**.008**	0.40 (0.14, 1.19)	.099	**0.79 (0.67, 0.93)**	**.004**	**8.34 (2.55, 27.33)**	**.001**
Drank alcohol								
No	Referent		Referent		Referent		Referent	
Yes	1.26 (0.96, 1.64)	.095	0.68 (0.44, 1.05)	.081	0.98 (0.93, 1.04)	.533	**2.13 (1.44, 3.15)**	**<.001**
Binge drink								
No	Referent		Referent		Referent		Referent	
Yes	**2.26 (1.41, 3.60)**	**.001**	0.53 (0.25, 1.11)	.093	0.91 (0.82, 1.00)	.054	**2.46 (1.27, 4.75)**	**.007**
Cannabis use								
No	Referent		Referent		Referent		Referent	
Yes	**2.26 (1.17, 4.39)**	**.016**	0.43 (0.17, 1.09)	.076	**0.86 (0.76, 0.98)**	**.022**	**6.86 (2.69, 17.52)**	**<.001**
Other drug use								
No	Referent		Referent		Referent		Referent	
Yes	2.08 (0.58, 7.45)	.260	**0.13 (0.02, 0.91)**	**.040**	0.79 (0.59, 1.05)	.100	**11.26 (1.48, 86.01)**	**.020**
Any gambling								
No	Referent		Referent		Referent		Referent	
Yes	1.01 (0.66, 1.55)	.950	0.74 (0.41, 1.33)	.318	0.97 (0.90, 1.06)	.532	**2.84 (1.57, 5.13)**	**.001**
Any self‐harm								
No	Referent		Referent		Referent		Referent	
Yes	0.98 (0.68, 1.41)	.896	0.77 (0.44, 1.35)	.367	1.07 (0.99, 1.15)	.106	1.39 (0.79, 2.44)	.260

*Note*: The *n*_analytic_ range varied from *n* = 9814 to *n* = 9870 for all models except for binge drinking (*n*_analytic_ range: 4357–4358), which had a smaller sample size because participants had to endorse ever drinking to be queried regarding binge‐drinking behaviors. Bolded findings are statistically significant at *p* < .05. Models adjusted for sex, age, race/ethnicity, pubertal status, internalizing symptoms, crystallized intelligence, household poverty, and educational/vocational attainment of the person most knowledgeable about the child.

Impulsivity was also associated with several risky health behaviors at age 14 (Table 3). Greater impulsivity was associated with an increased likelihood of being an occasional/regular cigarette smoker (OR = 3.15, 95% CI: 1.62, 6.13, *p* = .001), occasional/regular e‐cigarette/vaping user (OR = 2.74, 95% CI: 1.30, 5.75, *p* = .008), binge drinking (OR = 2.26, 95% CI: 1.41, 3.60, *p* = .001), and cannabis use (OR = 2.26, 95% CI: 1.17, 4.39, *p* = .016; Table 3). In contrast, better quality of decision‐making and risk adjustment were associated with a reduced likelihood of some health risk behaviors (e.g., quality of decision‐making and other drug use: OR = 0.13, 95% CI: 0.02, 0.91, *p* = .040; risk adjustment and cannabis use: OR = 0.86, 95% CI: 0.76, 0.98, *p* = .022). However, better risk adjustment was related to a reduced likelihood of all levels of cigarette and e‐cigarette/vaping use (e.g., occasionally/regularly) compared to nonsmokers and non‐vapers, respectively (Table 3). Drinking alcohol, gambling, and self‐harm were not statistically associated with impulsivity, quality of decision‐making, or risk adjustment (Table 3).

### Age 17 (longitudinal) models

Although many findings were attenuated in magnitude in the longitudinal (age 17) new onset health risk behavior models, risk‐taking and impulsivity remained consistent predictors of several health risk behaviors (Table 4). Similar to the previously reported cross‐sectional models, greater risk‐taking was associated with most forms of real‐life risk‐taking in adolescence (Table 4). Specifically, risk‐taking for reward at age 14 prospectively predicted the risk of cigarette use (e.g., occasional/regular smokers: OR = 3.24, 95% CI: 1.69, 6.24, *p* < .001), e‐cigarette and vaping use (e.g., occasional/regular vapers: OR = 2.45, 1.09, 5.49, *p* = .030), cannabis use (OR = 3.79, 95% CI: 2.26, 6.36, *p* < .001), other drug use (OR = 2.57, 95% CI: 1.20, 5.50, *p* = .016), and any gambling 3 years later at age 17 (OR = 2.43, 95% CI: 1.03, 5.74, *p* = .043; Table 4). Additionally, greater impulsivity at age 14 prospectively predicted greater risk of cigarette smoking (e.g., occasional/regular smokers: OR = 2.28, 95% CI: 1.51, 3.45, *p* < .001), and occasional/regular e‐cigarette/vaping use (OR = 2.08, 95% CI: 1.26, 3.42, *p* = .004), cannabis use (OR = 1.50, 95% CI: 1.02, 2.21, *p* = .039), and other drug use (OR = 1.97, 95% CI: 1.23, 3.14, *p* = .005). Unlike the cross‐sectional models, better decision‐making and risk adjustment were not statistically associated with any real‐life health risk behaviors (Table 4). Finally, drinking alcohol, binge drinking, and self‐harm were not prospectively predicted by any CGT measures (Table 4). There was no evidence of effect modification by sex in longitudinal models (Supporting Information S1: Table S3).

**TABLE 4:** Longitudinal associations between task measures of the Cambridge Gambling Task and health risk behaviors at age 17.

	Impulsivity		Quality of decision‐making		Risk adjustment		Risk‐taking	
	OR (95% CI)	*p*	OR (95% CI)	*p*	OR (95% CI)	*p*	OR (95% CI)	*p*
Cigarette smoking								
Nonsmokers	Referent		Referent		Referent		Referent	
Former smokers	**2.39 (1.26, 4.56)**	**.008**	1.06 (0.30, 3.70)	.932	0.91 (0.80, 1.03)	.143	**2.74 (1.07, 6.98)**	**.035**
Occasional/regular smokers	**2.28 (1.51, 3.45)**	**<.001**	0.70 (0.36, 1.33)	.272	0.92 (0.85, 1.00)	.057	**3.24 (1.69, 6.24)**	**<.001**
E‐cigarettes/vaping								
Nonvapers	Referent		Referent		Referent		Referent	
Former vapers	1.34 (0.70, 2.59)	.381	0.87 (0.36, 2.11)	.762	0.97 (0.85, 1.09)	.577	**4.10 (1.43, 11.76)**	**.009**
Occasional/regular vapers	**2.08 (1.26, 3.42)**	**.004**	1.15 (0.41, 3.26)	.789	0.97 (0.87, 1.08)	.538	**2.45 (1.09, 5.49)**	**.030**
Drank alcohol								
No	Referent		Referent		Referent		Referent	
Yes	1.01 (0.60, 1.70)	.980	0.48 (0.17, 1.35)	.164	0.94 (0.82, 1.09)	.424	1.21 (0.48, 3.05)	.681
Binge drink								
No	Referent		Referent		Referent		Referent	
Yes	1.02 (0.56, 1.84)	.959	1.43 (0.57, 3.61)	.444	1.10 (0.97, 1.25)	.128	1.07 (0.45, 2.51)	.886
Cannabis use								
No	Referent		Referent		Referent		Referent	
Yes	**1.50 (1.02, 2.21)**	**.039**	1.32 (0.78, 2.25)	.302	1.06 (0.99, 1.14)	.091	**3.79 (2.26, 6.36)**	**<.001**
Other drug use								
No	Referent		Referent		Referent		Referent	
Yes	**1.97 (1.23, 3.14)**	**.005**	1.02 (0.45, 2.32)	.963	0.97 (0.86, 1.08)	.540	**2.57 (1.20, 5.50)**	**.016**
Any gambling								
No	Referent		Referent		Referent		Referent	
Yes	1.49 (0.87, 2.55)	.147	1.24 (0.39, 3.93)	.716	0.95 (0.85, 1.07)	.420	**2.43 (1.03, 5.74)**	**.043**
Self‐harm								
None	Referent		Referent		Referent		Referent	
Non‐suicidal self‐harm	0.88 (0.50, 1.56)	.659	1.56 (0.69, 3.56)	.289	1.07 (0.97, 1.18)	.176	1.24 (0.63, 2.46)	.534
Suicide attempt	1.82 (0.88, 3.78)	.108	0.48 (0.13, 1.76)	.269	0.87 (0.72, 1.04)	.129	1.43 (0.20, 10.52)	.723

*Note*: After the removal of participants who positively endorsed the respective health risk behavior at age 14, the *n*_analytic_ range varied from *n* = 4532 to *n* = 7829 for all longitudinal models except for binge drinking (*n*_analytic_ range: 2639–2640), which had a smaller sample size because participants had to endorse ever drinking to be queried regarding binge‐drinking behaviors. Thus, the range of analytic sample sizes depended on the prevalence of the health risk behavior at age 14. Bolded findings are statistically significant at *p* < .05. Models adjusted for sex, age, race/ethnicity, pubertal status, internalizing symptoms, crystallized intelligence, household poverty, and educational/vocational attainment of the person most knowledgeable about the child.

## DISCUSSION

### Interpretation

Using a large cohort of adolescents representative of the UK population aged 14–17, we found that greater impulsivity and risk‐taking tendencies on a standardized gambling task are associated with and predict several adolescent health risk behaviors. In particular, risky betting was associated with all forms of self‐reported substance use and recent gambling at age 14. Risky betting, as measured by the CGT, was also a strong prospective predictor of adolescent risky behaviors at age 17. More specifically, while relationships were attenuated in magnitude, risky betting predicted all new‐onset substance use and gambling at age 17, except for alcohol use. None of the CGT subtasks were statistically related to adolescent self‐harm at age 14 or 17. To our knowledge, our findings are the first to link task‐based assessments of impulsivity and risk‐taking for reward with the development of health risk behaviors in a population‐based adolescent sample. Such insight is relevant for population‐level public health efforts that strive to curb negative health behaviors to prevent premature death and impairment.

Findings of the few most similar studies in adolescence (Hosozawa et al., 2021) and young adulthood (Goudriaan et al., 2011; Harvanko et al., 2013) are inconsistent. One found that poor performance on another decision‐making task (the Iowa Gambling Task), but not a response inhibition (impulsivity) task, was related to problematic alcohol use 2 years later in a college sample (Goudriaan et al., 2011). Another found that riskier decision‐making, as measured by the CGT, predicts short‐term at‐risk alcohol use (Harvanko et al., 2013), consistent with the present study. We observed relationships between risk‐taking and alcohol use, as measured by ever drinking alcohol and binge drinking, at age 14 but not later, at age 17. The same study (Harvanko et al., 2013) did not find that the CGT predicted pathological gambling or gambling frequency in their small community‐based sample (Harvanko et al., 2013). Notably, in the present study, gambling was more prevalent than other common substance use behaviors (e.g., binge drinking, cannabis use) at age 14. Furthermore, self‐reported gambling was statistically related to risk‐taking at both ages, underscoring the importance of this emerging public health issue (Armitage, 2021).

In terms of other work, the cross‐sectional conclusions of Grant et al. (2011) align with the present study in that risk‐taking may increase one's future risk of gambling in later adolescence. Using the CGT, Grant et al. (2011) observed that specific cognitive differences, such as riskier decision‐making, may predate psychopathological gambling behavior in emerging adulthood. At the same time, we should note that any of the aforementioned inconsistencies across studies may at least partly be attributable to differences in measurement operationalization. Different indices of decision‐making (i.e., Iowa Gambling Task vs. CGT) may be differentially related to health risk outcomes such as pathological gambling, and selective associations may help pinpoint explanatory processes (Wilson & Vassileva, 2018). For example, risk‐taking is not stable across measures (Holzmeister & Stefan, 2021; Pedroni et al., 2017). Differences in risk‐taking tendencies across methods may be influenced by changes to decision‐making strategies across measures (Pedroni et al., 2017). This insight may reveal which task‐based measures are the strongest predictors of real‐world risk‐taking and why. Finally, we should also note that some engagement in health risk behaviors may be considered a normative adolescent experience that may be a part of healthy development (Balogh et al., 2013). There may also be different levels of risk to health behaviors, with some serving a potentially adaptive function. For example, solitary but not social drinking may mediate the relationship between symptoms of depression and anxiety and later harmful drinking in young adulthood (Bilevicius et al., 2018).

In adolescence, decision‐making and risk‐taking processes are dependent upon areas of the brain undergoing marked cognitive development, potentially increasing youths' vulnerability to problematic or negative behaviors (Balogh et al., 2013; Casey et al., 2008). For example, an affective responsiveness‐executive control imbalance may influence adolescent decision‐making (Balogh et al., 2013), while individual‐level neural differences in reward responsiveness may explain adolescent risk‐taking behaviors (Casey et al., 2008). Without specific neurobiological insight from our study participants (e.g., neuroimaging) to guide our interpretation, we observed that a greater risk‐taking propensity at age 14 was reflective of engagement in concurrently measured health risk behaviors and an indicator of future risk of engagement in substance use and gambling behavior 3 years later. Greater risk‐taking could be a useful indicator of real‐time adverse health experiences in adolescence. Additionally, impulsivity, a construct central to substance and behavioral addictions (Lee et al., 2019), materialized as a potentially reliable secondary predictor of health risk behaviors. Though the relationships that persisted between impulsivity and substance use and gambling varied according to adolescent age, impulsivity predicted cannabis use at both ages. Evidence supporting a relationship with problematic cannabis use is limited (Lee et al., 2019). We may not have observed some relationships between impulsivity and other health outcomes because of operationalization differences between the CGT and other widely used measures. For example, impulsivity captured by delay discounting measures (Kirby et al., 1999) may be more relevant to substance use (Argyriou et al., 2018). Distinctions in the facets of impulsivity most pertinent to certain health risk behaviors may also explain our null self‐harm findings.

While differences have been observed in performance on the CGT in case‐control studies of young people who self‐harm (McHugh et al., 2019), a recent experimental study examining behavioral measures of impulsiveness among adults who attempted suicide, experienced suicidal ideation, and controls observed no between‐group differences on any behavioral measures (Millner et al., 2020). However, those who attempted suicide self‐reported higher negative urgency impulsivity (an emotionally‐driven rash or regrettable [impulsive] response to alleviate distress) (Whiteside & Lynam, 2001) in one substudy (Millner et al., 2020). The authors speculate that when experiencing negative affect and surrounding a suicide attempt, impulsivity may be heightened compared to those who experience suicidal ideation, but that there may be no group differences in overall trait impulsiveness, consistent with work challenging dominant theories of suicidality (Millner et al., 2020). In fact, self‐report trait impulsivity among adolescents has not reliably predicted future self‐harm in longitudinal studies (Garisch & Wilson, 2015; Lockwood et al., 2020), including among those systematically summarized (Lockwood et al., 2017). Cross‐sectionally, self‐report of negative urgency impulsiveness repeatedly demonstrates associations with self‐injurious thoughts and behaviors (Lockwood et al., 2017, 2020; Millner et al., 2020). As outlined by Lockwood and colleagues (Lockwood et al., 2017), negative urgency may affect one's propensity to engage in easily accessible maladaptive distress reduction methods (e.g., self‐harm) to regulate their mood state (Cyders & Smith, 2008). Successful use of this method may lead to negative reinforcement and maintenance (Klonsky, 2007). This theoretical model may be well supported in adolescent samples where the means to regulate negative mood states in a time‐sensitive manner is best facilitated with quickly available solutions for young persons under the age of majority for whom other options (e.g., substances) may be harder to procure. Ecological momentary assessment using validated indicators of impulsivity, risk‐taking, and mood states may better delineate differences between adolescents who self‐harm versus not. How standardized assessment of impulsivity relates to adolescent self‐harm remains unclear (McHugh et al., 2019).

To conclude, better risk adjustment and quality of decision‐making were inconsistently related to health risk behaviors at age 14, and not at all at age 17. One trend that persisted cross‐sectionally (age 14) was an association between better risk adjustment and a reduced likelihood of any concurrent cigarette or e‐cigarette vaping/use. This pattern suggests that better risk adjustment on a task‐based measure, defined here as the tendency for the youth to adjust their bet dependent on the odds, is a feature of decision‐making potentially relevant to adolescent consumption of certain harmful substances with well‐documented negative health consequences (Marques et al., 2021).

### Strengths and limitations

Study strengths include the large population‐based sample and application of survey weights to draw population‐level inferences, the 3‐year follow‐up period, the computerized task‐based exposure, and the initial oversampling of disadvantaged families and ethnic minorities. Families that remain in population‐based surveys tend to come from more affluent backgrounds and be of white ethnicity. The oversampling approach used for the MCS increases confidence that, over the long term, the sample is representative of the original population.

Study‐specific limitations include that at age 17, gambling was measured via an online survey after the two‐step in‐home assessment (CGT/survey). Requiring participation in a third survey step explains the smaller sample size for the longitudinal gambling outcome. Social desirability bias could have impacted findings because adolescents answered most health risk behavior questions with study interviewers present. We would expect any under‐reporting to underestimate the magnitude of associations. Similarly, those lost to follow‐up exhibited poorer decision‐making ability than those retained, potentially weakening relationships. At ages 14/17, participants were asked about past‐year self‐harm and at age 17, they were also queried about attempting suicide. We distinguished between non‐suicidal self‐harm and suicide attempt at age 17. However, we would have preferred the same questions at both ages. Having a combined age 14 self‐harm category may have obscured differences in decision‐making and risk‐taking by self‐harm type, should they exist. Although, since we did not observe any self‐harm relationships, it is unlikely.

It is important to highlight further that as participants aged, there were some differences in question phrasing and answer options, a possible result of ensuring developmentally acceptable questions and capturing contemporary health risk behaviors across time. For example, at age 17 but not age 14, participants were asked about e‐cigarette use and vaping and had greater answer options (e.g., “never tried”, “ever tried… once”). The expansion of the survey item to include vaping at age 17 may reflect increasing use in the population‐at‐large and alignment between the survey and monitoring of public health areas of interest. In contrast, the different answer options may reflect the potential for greater variability in health risk patterns as adolescents age and are exposed to more health risk situations through their daily life, including interactions with their peers. We expect any misclassification in our cross‐sectional models to result in a bias toward the null (i.e., underestimated). Some adolescents who vaped at age 14 may be classified as new‐onset vapers at age 17 because they were not surveyed on earlier vaping use, only e‐cigarette use. To explore the potential impact on our study findings, we ran sensitivity analyses (data not shown), adjusting for age 14 vaping use, when predicting future (age 17) use, not limited to new onset users. All but one relationship statistically held; risk‐taking no longer predicted age 17 occasional/regular vaping use.

## CONCLUSION

Using a prospective cohort study, we demonstrate that task‐based measures of impulsivity and risk‐taking may hold utility as markers of future substance use and gambling behavior among adolescent populations. Indicators of better decision‐making do not appear to be useful long‐term predictors of health risk behaviors in a large population‐based sample of adolescents undergoing rapid cognitive development. In particular, a tendency for risk‐taking, as measured by an easily administered task‐based measure, may be helpful in the development of adolescent‐targeted indicated prevention efforts to reduce negative life experiences associated with harmful substances and behaviors, such as drug use and gambling.

## AUTHOR CONTRIBUTIONS

**Nicole G. Hammond:** Conceptualization; formal analysis; methodology; project administration; supervision; validation; writing—original draft; writing—review and editing. **Georgia Condran:** Conceptualization; writing—review and editing. **Elise E. DeVito:** Conceptualization; methodology; writing—review and editing. **Marie‐Claude Geoffroy:** Methodology; writing—review and editing. **Ian Colman:** Conceptualization; methodology; project administration; supervision; validation; writing—review and editing.

## CONFLICT OF INTEREST STATEMENT

The authors declare no conflicts of interest.

## ETHICAL CONSIDERATIONS

The University of Ottawa Research Ethics Board (File #H‐11‐21‐7644) approved this study (November 18, 2021). Adolescent and parent/caregiver participants of the Millennium Cohort Study provided informed consent.

## Supporting information

Supporting Information S1

## Data Availability

Data is publicly available to users with authorized projects through the UK Data Service (ukdataservice.ac.uk).
