# Supplementary material for: Decision‐making and risk‐taking as predictors of health risk behaviors in the Millennium Cohort Study
Source: JCPP Adv. 2025 Dec 9:e70071. Online ahead of print. doi: 10.1002/jcv2.70071 (PMC13338997; doi:10.1002/jcv2.70071)
Supplement: Supplementary file 1 — Supporting Information S1 [file JCV2-9999-e70071-s001.docx]

**Decision-making and risk-taking as predictors of health risk behaviours in the Millennium Cohort Study**

**Supporting Information**

**APPENDIX S1.**

**eMethods**

Below, we present additional information on the outcomes and confounders.

***Risky health behaviours (outcomes)***

Our study outcomes (substance use, gambling, and self-harm) were collected as part of youth self-report risky behaviour modules administered in 2015 (age 14) and 2018 (age 17). Additional information on the question wording and coding of risky health behaviours is provided in **Table S1**.

***Substance use.*** For ***cigarette smoking,*** we categorized participants as never smokers (tried smoking ≤1 time, referent), former smokers (used to smoke but not now), occasional (sometimes smoke, but <1 per week) and/or regular smokers (≥1 per week). We combined occasional and regular smokers into one category to ensure adequate cell sizes for analyses and to mitigate concerns regarding different answer options at ages 14 versus 17 years (please see Appendix S2 for the exact survey questions and answers). The smoking question posed to participants was identical at 14 and 17 years. ***E-cigarette use/vaping*** was operationalized similarly. However, due to some differences, including an expanded number of answer options at 17 years, our outcome operationalization slightly differed in cross-sectional and prospective models. We classified respondents as nonvapers (age 14: never tried or used, referent; age 17: tried an e-cigarette or vaping device ≤1 time, referent), former vapers (used to use an e-cigarette or vaping device but don’t now), occasional vapers (age 14: occasionally but not every day; age 17: sometimes vaper, but <1 per week) and/or regular vapers (age 14: every day user; age 17: ≥1 per week). Consistent with our approach to cigarette use, we combined occasional and regular e-cigarette/vaper users into one occasional-regular user category at ages 14 and 17 years. For ***binge drinking,*** participants were first asked whether they had ever consumed more than a few sips of an alcoholic drink (i.e., ever ***drank alcohol*** [yes/no (referent)]). If yes at 14/17 years, they were subsequently asked to report whether they “ever had five or more alcoholic drinks at a time? A drink is half a pint of lager, beer or cider, one alcopop, a small glass of wine, or a measure of spirits” (yes/no [referent]). Participants were asked to report whether they had ever tried ***cannabis*** or ***other illegal drugs*** (e.g., ecstasy, cocaine, speed) at 14/17 years. At age 14, respondents indicated whether they had used cannabis (yes/no [referent]) or any other (non-cannabis) illegal drug (yes/no [referent]). In contrast, at age 17, respondents were presented with a list of illicit drugs (cannabis, cocaine powder, acid or LSD, ecstasy, heroin, crack, speed or amphetamines, semeron, ketamine, mephedrone, and psychoactive substances) and asked to report whether they had tried each (yes/no). We removed respondents who reported consuming “semeron” (n=5), a fictitious drug, or who did not respond to the question (n=25). We retained cannabis as a separate drug use category because it is legal for adults in some countries and combined the non-cannabis illicit drugs into an “other” drug use category.

***Gambling.*** At both time points (14/17 years), participants were asked, “Have you spent any of your **own money** on any of the following in the past 4 weeks? We want to know about games you played yourself.” Participants were prompted to report whether they used “fruit machines”, placed “a private bet for money (e.g., with friends)”, placed “a bet at a betting shop (e.g., on football or horseracing)”, or engaged in “any other gambling”. At age 17, an example of other (“online gambling”) was given. We classified respondents as having engaged in gambling at either time point if they positively endorsed one or more of the four forms of gambling, versus not (referent).

***Self-harm.*** At 14 and 17 years, adolescents self-reported self-harm behaviours. At 14 years, respondents were asked about nonspecific (with or without suicidal intent) self-harm: “In the past year have you hurt yourself on purpose in any way?” (yes/no [referent]). At age 17, participants were again asked about nonspecific self-harm, but the question-and-answer options were more developmentally advanced: “During the last year, have you hurt yourself on purpose in any of the following ways?” (yes/no for each): “cut or stabbed yourself”, “burned yourself”, “bruised or pinched yourself”, “taken an overdose of tablets”, “pulled out your hair”, and “hurt yourself some other way”. In addition, at 17 years, participants were asked about self-harm with suicidal intent: “Have you ever hurt yourself on purpose in an attempt to end your life?” (yes/no). Therefore, in longitudinal models, we were able to differentiate between non-suicidal and suicidal self-harm. Respondents who positively endorsed any form of past-year self-harm and ever self-harm with suicidal intent were classified as having made a suicide attempt (self-harm with suicidal intent). Respondents who positively endorsed any form of past-year self-harm but never made a suicide attempt were considered to have engaged in non-suicidal self-harm. Together, we used this information to create a three-level self-harm measure at 17 years: no self-harm (referent), non-suicidal self-harm, and (ever) suicide attempt.

**Confounders**

We selected confounders known to be associated with the exposures and/or outcomes. For the child, we included sex (female/male [referent]), age (continuous), race/ethnicity, pubertal status, internalizing symptoms, and crystallized intelligence. The Millennium Cohort Study captured race/ethnicity as a six-level derived variable, which we recoded by retaining the three most prevalent categories (White [referent], Mixed, Pakistani and Bangladeshi) and collapsing the remaining ethnicities into an “Other” category (Indian, Black or Black British, and Other [including Chinese]) for sufficient sample sizes in each category. We computed a measure of internalizing symptoms (depression and anxiety, range: 0-20) by combining the emotional symptoms and peer problems subscales of the Strengths and Difficulties Questionnaire,^4^ completed by the person most knowledgeable on the child (most often the biological mother).^5^ The shortened (20-item) version of the Applied Psychology Unit (APU) Vocabulary Test^6^ measured crystallized intelligence. Participants were presented with 20 words individually. Following the presentation of each word, the participant was shown a (new) list of 5 words and asked to pick the initially presented word’s synonym.^7^ Participants were allocated a total of 4 minutes, and the test increased in complexity as it progressed.^7^ A total score of 0-20 is given, with a maximum of 1 point per correct answer. Pubertal status was assessed using the Pubertal Development Scale.^8^ Participants were asked to report on the presence of 5 pubertal milestones (rated as 1 [absence] to 4 [completion]): body hair, growth spurt, skin changes, breast growth (females), facial hair (males), menstruation (females), and voice changes (males). We computed an average of the five items for each sex, prorated for missingness (range: 1-4).

At the family level, we adjusted for household poverty and the educational and vocational attainment of the person most knowledgeable about the child. The household poverty indicator used here was a Millennium Cohort Study-derived variable. Families were classified as living in poverty if the weekly household income was <60% of the median household income for the United Kingdom, based on the Organisation for Economic Co-operation and Development equivalencies.^5^ The highest academic and vocational qualifications of the person most knowledgeable about the child were captured according to National Vocational Qualification (NVQ) standards. We created a three-level measure by collapsing NVQ levels 1-3 (e.g., General Certificate of Secondary Education levels), 4-5 (e.g., BA degree+), and other categories (e.g., overseas qualifications only).

**References**

1. Atkinson M. Millennium Cohort Study: Interpreting the CANTAB Cognitive Measures (First Edition). https://cls.ucl.ac.uk/wp-content/uploads/2017/07/mcs5_cantab_assessments_

data_note.pdf. Published January, 2015: Accessed February 12, 2024.

2. DeVito EE, Blackwell AD, Kent L, et al. The effects of methylphenidate on decision making in attention-deficit/hyperactivity disorder. *Biol Psychiatry*. 2008;64(7):636-639. doi:10.1016

/j.biopsych.2008.04.017

3. Wilson MJ, Vassileva J. Decision-making under risk, but not under ambiguity, predicts pathological gambling in discrete types of abstinent substance users. *Front Psychiatry*. 2018;9:239. doi:10.3389/fpsyt.2018.00239

4. Goodman R. The Strengths and Difficulties Questionnaire: a research note. *J Child Psychol Psychiatry*. 1997;38(5):581-586. doi:10.1111/j.1469-7610.1997.tb01545.x

5. Fitzsimons E. Millennium Cohort Study Sixth Survey 2015-2016: User Guide (First Edition). https://cls.ucl.ac.uk/wp-content/uploads/2018/10/mcs6_user_guide_28march2017.pdf. Published February, 2017. Accessed February 14, 2024.

6. Closs SJ, Hutchings MJ. *APU Arithmetic Test*. London: Hodder and Stoughton; 1976.

7. Moulton V, McElroy E, Richards M, et al. A Guide to the Cognitive Measures in Five British Birth Cohort Studies. https://www.closer.ac.uk/wp-content/uploads/250820-Guide-to-cognitive-measures-in-five-British-birth-cohorts.pdf. Published August, 2020. Accessed February 14, 2024.

8. Petersen AC, Crockett L, Richards M, Boxer A. A self-report measure of pubertal status: reliability, validity, and initial norms. *J Youth Adolesc*. 1988;17(2):117-133. doi:10.1007/BF01537962

| **APPENDIX S2** | | |
| --- | --- | --- |
| **Table S1.** Breakdown of the coding of adolescent risky health behaviours (study outcomes) at ages 14 and 17. Survey questions are from the Millennium Cohort Study, Sweeps 6 (2015) and 7 (2018). | | |
| **Outcomes** | **Age 14**^1^ | **Age 17**^2,3^ |
| **Cigarette smoking** | “Please read the following statements carefully and decide which one best describes you. Do not include electronic cigarettes (e-cigarettes).”   - “I have never smoked cigarettes” (never smoker) - “I have only ever tried smoking cigarettes once” (never smoker) - “I used to smoke sometimes but I never smoke a cigarette now” (former smoker) - “I sometimes smoke cigarettes now but I don’t smoke as many as one a week” (occasional/regular smoker) - “I usually smoke between one and six cigarettes a week” (occasional/regular smoker) - “I usually smoke more than six cigarettes a week” (occasional/regular smoker) | “Please read the following statements carefully and decide which ONE best describes you. Do not include electronic cigarettes (e-cigarettes).”   - “I have never smoked cigarettes” (never smoker) - “I have only ever tried smoking cigarettes once” (never smoker) - “I used to smoke sometimes but I never smoke a cigarette now” (former smoker) - “I sometimes smoke cigarettes now but I don’t smoke as many as one a week” (occasional smoker) - “I usually smoke between one and six cigarettes a week” (regular smoker) - “I usually smoke more than six cigarettes a week” (regular smoker) |
| **E-cigarettes/**  **vaping** | “Please read the following statement carefully and decide which one best describes you.”   - “I've never used or tried electronic cigarettes (e-cigarettes)” (nonuser) - “I have used e-cigarettes but don't at all now” (former user) - “I now smoke e-cigarettes occasionally but not every day” (occasional/regular user) - “I smoke e-cigarettes every day” (occasional/regular user) | “Please read the following statements carefully and decide which ONE best describes you.”   - “I have never tried an e-cigarette or vaping device” (nonvaper) - “I have only ever tried an e-cigarette or vaping device once” (nonvaper) - “I used to use an e-cigarette or vaping device sometimes but I never use an e-cigarette or vaping device now” (former vaper) - “I sometimes use an e-cigarette or vaping device now but I don’t use an e-cigarette or vaping device as often as one a week” (occasional vaper) - “I usually use an e-cigarette or vaping device between one and six times a week” (regular vaper) - “I usually use an e-cigarette or vaping device more than six times a week” (regular vaper) |
| **Drank alcohol** | “Have you ever had an alcoholic drink? That is more than a few sips.”   - “Yes” - “No” | “Have you ever had an alcoholic drink? That is more than a few sips. A drink is half a pint of lager, beer or cider, one alcopop, a small glass of wine, or a measure of spirits.”   - “Yes” - “No” |
| **Binge drink** | “Have you ever had five or more alcoholic drinks at a time? A drink is half a pint of lager, beer or cider, one alcopop, a small glass of wine, or a measure of spirits.”   - “Yes” - “No” | “Have you ever had five or more alcoholic drinks at a time? A drink is half a pint of lager, beer or cider, one alcopop, a small glass of wine, or a measure of spirits.”   - “Yes” - “No” |
| **Cannabis use** | “Have you ever tried any of the following things?” “Cannabis (also known as weed, marijuana, dope, hash or skunk)?”   - “Yes” - “No” | “The next few questions are about drugs, not including cigarettes and alcohol or prescribed medication. Have you ever taken any of the following?”  “Cannabis (also called Marijuana, Dope, Pot, Blow, Hash, Skunk, Puff, Grass, Draw, Ganja, Spliff, Smoke, Weed)”   - “Yes” - “No” |
| **Other drug use** | “Have you ever tried any of the following things?”  “Any other illegal drug (such as ecstasy, cocaine, speed)?”   - “Yes” - “No” | “The next few questions are about drugs, not including cigarettes and alcohol or prescribed medication. Have you ever taken any of the following?”   - “Cocaine powder (also called Coke, Charlie, ‘C’, Snow, Percy, Toot)” “Yes/No” - “Acid or LSD (also called Acid, Trips, Dots, Flash, Smilies)” “Yes/No” - “Ecstasy (also called ‘E’, MDMA, Molly, Mitsubishis, Rolex’s, Dolphins, XTC)” “Yes/No” - “Heroin (also called Brown, Smack, Skag, Horse, Gear, ‘H’)” “Yes/No” - “Crack (also called Rocks, Stones, Freebase, Wash)” - “Speed or Amphetamines, (also called Whizz, Sulphate, Billy)” “Yes/No” - “Methamphetamine (also called Crystal meth, dexies, chalk, and ice)” “Yes/No” - “Ketamine (also called Green, ‘K’, super K)” “Yes/No” - “Mephedrone (also called Meow Meow, M-Cat, Bubble, Charge, Drone, 4MMC)” “Yes/No” - “Psychoactive substances (such as salvia, spice, trance, schrooms)” “Yes/No”   If yes to any of the above, classified as having used other drugs. |
| **Any gambling** | “Have you spent any of YOUR OWN money on any of the following in the past 4 weeks? We want to know about games you played yourself.”   - “Fruit machines?” “Yes/No” - “Placing a private bet for money (e.g. with friends)?” “Yes/No” - “Placing a bet at a betting shop (e.g. on football or horseracing)?” “Yes/No” - “Any other gambling?” “Yes/No”   If yes to any of the above, classified as having gambled. | “Have you spent any of your **own money** on any of the following in the past 4 weeks? We want to know about games you played yourself.”   - “Fruit machines?” “Yes/No” - “Placing a private bet for money (e.g. with friends)?” “Yes/No” - “Placing a bet at a betting shop (e.g. on football or horseracing)?” “Yes/No” - “Any other gambling (e.g. online gambling)?” “Yes/No”   If yes to any of the above, classified as having gambled. |
| **Self-harm** | “In the past year have you hurt yourself on purpose in any way?”   - “Yes” - “No” | Nonspecific self-harm: “During the last year, have you hurt yourself on purpose in any of the following ways? Please select one answer on every row”   - “Cut or stabbed yourself” “Yes/No” - “Burned yourself” “Yes/No” - “Bruised or pinched yourself” “Yes/No” - “Taken an overdose of tablets” “Yes/No” - “Pulled out your hair” “Yes/No” - “Hurt yourself some other way” “Yes/No”   If yes to any of the above, classified as having self-harmed.  Suicide attempt: “Have you ever hurt yourself on purpose in an attempt to end your life?”   - “Yes’” - “No”   If yes to nonspecific self-harm and suicide attempt, were classified as having made a suicide attempt.  If yes to nonspecific self-harm but no to a suicide attempt, were classified as having engaged in non-suicidal self-harm. |

**References**

1. Centre for Longitudinal Studies, UCL Institute of Education. Millennium Cohort Study Sweep 6 Young Person Questionnaire. https://cls.ucl.

ac.uk/wp-content/uploads/2017/12/MCS6-Young-Person-Questionnaire.pdf. Accessed February 14, 2024.

2. Centre for Longitudinal Studies, UCL Institute of Education. Millennium Cohort Study Seventh Sweep (MCS7) Age 17 Survey: Young Person Self-Completion Questionnaire. https://cls.ucl.ac.uk/wp-content/uploads/2020/01/MCS7-Young-Person-Self-Completion-Questionnaire.pdf. Published January 2020. Accessed February 14, 2024.

3. Centre for Longitudinal Studies, UCL Institute of Education. Millennium Cohort Study Seventh Sweep (MCS7) Age 17 Survey: Young Person Online (CAWI) Questionnaire. https://cls.ucl.ac.uk/wp-content/uploads/2020/02/MCS7-Young-Person-Online-CAWI-Questionnaire.pdf. Published January, 2020. Accessed February 14, 2024.

| **Table S2.** Likelihood ratio tests to assess improvement in model fit with the inclusion of a sex by exposure (impulsivity, risk-taking) interaction term in the multinomial and logistic cross-sectional regression models. | | | | | | |
| --- | --- | --- | --- | --- | --- | --- |
|  | **Impulsivity** | | | **Risk-taking** | | |
|  |  | Likelihood ratio test | |  | Likelihood ratio test | |
|  | Interaction *P*-value | χ^2^ (df) | Improvement in model fit *p*-value | Interaction *P*-value | χ^2^ (df) | Improvement in model fit *p*-value |
| Cigarette smoking |  | **17.51 (2)** | **<0.001** |  | **12.52 (2)** | **0.002** |
| Nonsmokers | Referent |  |  | Referent |  |  |
| Former smokers | **0.029** |  |  | 0.575 |  |  |
| Occasional/regular smokers | **0.040** |  |  | **0.028** |  |  |
| E-cigarettes/vaping |  | **11.94 (2)** | **0.003** |  | **12.15 (2)** | **0.002** |
| Nonvapers | Referent |  |  | Referent |  |  |
| Former vapers | 0.638 |  |  | 0.536 |  |  |
| Occasional/regular vapers | **0.012** |  |  | **0.016** |  |  |
| Drank alcohol |  | -- | -- |  | -- | -- |
| No | Referent |  |  | Referent |  |  |
| Yes | 0.362 |  |  | 0.095 |  |  |
| Binge-drink |  | -- | -- |  | **8.13 (1)** | **0.004** |
| No | Referent |  |  | Referent |  |  |
| Yes | 0.289 |  |  | **0.035** |  |  |
| Cannabis use |  | -- | -- |  | **22.48 (1)** | **<0.001** |
| No | Referent |  |  | Referent |  |  |
| Yes | 0.879 |  |  | **0.001** |  |  |
| Other drug use |  | -- | -- |  | **9.33 (2)** | **0.002** |
| No | Referent |  |  | Referent |  |  |
| Yes | 0.585 |  |  | **0.011** |  |  |
| Any gambling |  | -- | -- |  | -- | -- |
| No | Referent |  |  | Referent |  |  |
| Yes | 0.679 |  |  | 0.562 |  |  |
| Any self-harm |  | -- | -- |  | -- | -- |
| No | Referent |  |  | Referent |  |  |
| Yes | 0.938 |  |  | 0.538 |  |  |
| ***Note.*** Likelihood ratio tests were conducted when there was a statistically significant (*p* < 0.05) interaction term (see Interaction *p*-value columns). Bolded findings are statistically significant at *p* < 0.05. Models adjusted for sex, age, race/ethnicity, pubertal status, internalizing symptoms, crystallized intelligence, household poverty, and educational/vocational attainment of the person most knowledgeable about the child. | | | | | | |

| **Table S3.** Likelihood ratio tests to assess improvement in model fit with the inclusion of a sex by exposure (impulsivity, risk-taking) interaction term in the multinomial and logistic longitudinal regression models. | | | | | | |
| --- | --- | --- | --- | --- | --- | --- |
|  | **Impulsivity** | | | **Risk-taking** | | |
|  |  | Likelihood ratio test | |  | Likelihood ratio test | |
|  | Interaction *P*-value | χ^2^ (df) | Improvement in model fit *p*-value | Interaction *P*-value | χ^2^ (df) | Improvement in model fit *p*-value |
| Cigarette smoking |  | -- | -- |  | -- | -- |
| Nonsmokers | Referent |  |  | Referent |  |  |
| Former smokers | 0.932 |  |  | 0.506 |  |  |
| Occasional/regular smokers | 0.590 |  |  | 0.561 |  |  |
| E-cigarettes/vaping |  | -- | -- |  | -- | -- |
| Nonvapers | Referent |  |  | Referent |  |  |
| Former vapers | 0.250 |  |  | 0.847 |  |  |
| Occasional/regular vapers | 0.844 |  |  | 0.312 |  |  |
| Drank alcohol |  | **7.71 (1)** | **0.005** |  | -- | -- |
| No | Referent |  |  | Referent |  |  |
| Yes | **0.024** |  |  | 0.841 |  |  |
| Binge-drink |  | **11.97 (1)** | **<0.001** |  | -- | -- |
| No | Referent |  |  | Referent |  |  |
| Yes | **0.005** |  |  | 0.902 |  |  |
| Cannabis use |  | -- | -- |  | -- | -- |
| No | Referent |  |  | Referent |  |  |
| Yes | 0.653 |  |  | 0.278 |  |  |
| Other drug use |  | -- | -- |  | -- | -- |
| No | Referent |  |  | Referent |  |  |
| Yes | 0.762 |  |  | 0.189 |  |  |
| Any gambling |  | -- | -- |  | -- | -- |
| No | Referent |  |  | Referent |  |  |
| Yes | 0.711 |  |  | 0.972 |  |  |
| Self-harm |  | -- | -- |  | -- | -- |
| None | Referent |  |  | Referent |  |  |
| Non-suicidal self-harm | 0.984 |  |  | 0.122 |  |  |
| Suicide attempt | 0.973 |  |  | 0.078 |  |  |
| ***Note.*** Likelihood ratio tests were conducted when there was a statistically significant (*p* < 0.05) interaction term (see Interaction *p*-value columns). Bolded findings are statistically significant at *p* < 0.05. Models adjusted for sex, age, race/ethnicity, pubertal status, internalizing symptoms, crystallized intelligence, household poverty, and educational/vocational attainment of the person most knowledgeable about the child. | | | | | | |
